# Rapid High Performance Liquid Chromatography Determination and Optimization of Extraction Parameters of the α-Asarone Isolated from *Perilla frutescens* L.

**DOI:** 10.3390/molecules22020270

**Published:** 2017-02-10

**Authors:** Seung Hwan Hwang, Shin Hwa Kwon, Young-Hee Kang, Jae-Yong Lee, Soon Sung Lim

**Affiliations:** 1 Department of Food Science and Nutrition, Hallym University, 1 Hallymdeahak-gil, Chuncheon 24252, Korea; isohsh@gmail.com (S.H.H.); yhkang@hallym.ac.kr (Y.-H.K.); 2 Institute of Korean Nutrition, Hallym University, 1 Hallymdeahak-gil, Chuncheon 24252, Korea; 3 Institute of Natural Medicine, Hallym University, 1 Hallymdeahak-gil, Chuncheon 24252, Korea; ksh8976@nate.com (S.H.K.); jyolee@hallym.ac.kr (J.-Y.L.); 4 Department of Biochemistry, School of Medicine, Hallym University, 1 Hallymdeahak-gil, Chuncheon 24252, Korea

**Keywords:** *Perilla frutescens *L., α-asarone, validation, optimization, response surface methodology, HPLC

## Abstract

Response surface methodology (RSM), based on a central composite design, was used to determine the best liquid-to-raw material ratio (10:3–15 mL/g), extraction time (1–3 h), and ethanol concentration (50%–100%) for maximum content of α-asarone from *Perilla frutescens* (PF) extract. Experimental values of α-asarone were 9.51–46.36 mg/g; the results fitted a second-order quadratic polynomial model and correlated with the proposed model (R^2^ > 0.9354). The best conditions were obtained with extraction time of 1.76 h, liquid-to-raw material ratio of 10:13.5 mL/g, and ethanol concentration of 90.37%. Under these conditions, the model predicted extraction content of 40.56 mg/g, while experimental PF content of α-asarone was 43.84 mg/g dried plant. Optimized conditions determined for maximum content of α-asarone were similar to the experimental range. Experimental values agreed with those predicted, thus validating and indicating suitability of both the model and the RSM approach for optimizing extraction conditions. In addition, a reliable, reproducible and accurate method for the quantitative determination of α-asarone by High Performance Liquid Chromatography (HPLC) analysis was developed with limit of detection (LOD), limit of quantitation (LOQ) values of 0.10 and 0.29 µg/mL and excellent linearity (R^2^ > 0.9999).

## 1. Introduction

Response surface methodology (RSM) is a useful statistical technique for evaluating the effects and interactions of multiple factors. RSM can also be effective in finding the combination of factors that produce the optimum response [1]. The main advantage of this technology is that fewer experimental trials are needed to evaluate multiple factors, and their interactions, making it less laborious and time-consuming than other optimization techniques (e.g., the “one-variable-at-a-time” optimization) [2]. Recently, He et al. optimized the protein yield, total phenolic contents (TPC), and lecithin content of black turtle bean extract using RSM [3]. In another study, researchers found optimum conditions for antioxidant activity of *Artemisia absinthium* with RSM [4], Rahmani et al. reported on rapid quantitative determination and optimization of extraction condition of deoxynivalenol in rice samples using HPLC and RSM [5]. In addition, D’Archivio et al. investigated the effects of molecular structure and mobile phase characteristics on RP-HPLC of s-triazines under linear gradient-elution conditions and optimized the eluent composition and the slope of the linear gradient by RSM [6]. D’Archivio et al. also reported the development and validation of a high-performance liquid chromatography (UHPLC) method for the simultaneous detection of seven non-steroidal anti-inflammatory drugs (NSAIDs) in dialyzed samples and optimized the UHPLC separation of NSAIDs using RSM [7].

The central composite design (CCD) is compatible with response surface methods, allowing rapid screening of a wide range of conditions while also indicating the role of each factor [8]. Various validation methods were used for the analysis of the constituents from natural products including HPLC ultraviolet (UV) detector [9], mass spectrometry (MS) [10], and UHPLC UV and MS [11], etc. Among them, the most commonly used method is HPLC. The HPLC method has been described for the simultaneous determination of polyphenol and flavonoids in natural products [12]. In addition, drug concentrations in rat or human plasma in the recent literature are reported by HPLC [13]. The HPLC method is economical, involves a simple extraction procedure, requires lesser sample volume and possesses a shorter chromatographic analysis run time as compared to other reported methods.

*Perilla frutescens* L. (PF) is a traditional Chinese medicinal herb that has been used in China for centuries to treat various diseases including depression, anxiety, tumor, cough, bacterial, fungal infections and allergy [14]. The recent work of our research team reported that PF extract showed enhanced cholesterol efflux from oxidized low density lipoprotein (LDL)-exposed macrophases and α-asarone was isolated from PF and characterized as a major component enhancing the induction of adenosine triphosphate (ATP)-binding cassette sub-family A member 1 and ATP-binding cassette sub-family G member 1 in macrophages exposed to oxidized LDL [15]. α-Asarone is found in diverse vegetables including Araceae plants (*Acorus*) and Annonnaceae tree (*Guatteriagaumeri*). Natural product extracts containing α-asarone have been used to elicit anti-epileptic and neurological, diuretic, hypocholesterolemic, and anti-cholelithiasis effects in Indian, Korean and Mexican traditional medicine [16,17]. According to another study, α-asarone has similar activity to the hepatic 3-hydroxy-3-methylglutaryl-coenzyme A reductase (HMG-CoA reductase) [18].

To date, however, there have been no studies of the development and method validation of a simple and reliable method for identification and quantification of α-asarone in PF using by HPLC method and to optimize the extraction procedures with RSM. In this study, we described the development and method validation of a simple and reliable method for identification and quantification of α-asarone from PF using by HPLC method and to optimize the extraction procedures for the ratio of liquid to raw material, extraction time and solvent concentration.

## 2. Results and Discussion

### 2.1. Structural Determination of Isolate Compounds

**Figure 1:**
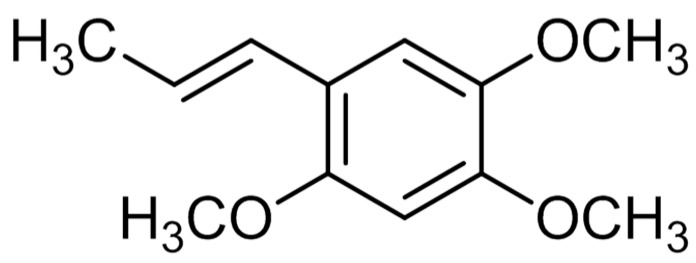
The structure of α-asarone.

The α-asarone was separated from 80% ethanol extract by silicagel column chromatography to obtain 19.4 mg. This compound was identified by comparing their ^1^H- and ^13^C-NMR spectra and NMR correlation spectra such as correlation spectroscopy (COSY), heteronuclear multiple bond correlation (HMBC) and heteronuclear multiple quantum correlation (HMQC) with previously reported data (Figure 1).

### 2.2. Linearity

Validation parameters included linearity, sensitivity, accuracy, precision, specificity, and stability. Serially diluted solutions of the α-asarone prepared in the range of 1, 10, 25, 50 and 100 µg/mL was injected into the HPLC, and calibration curve equations were calculated. The validation parameters of the calibration curve were shown in Table 1. Excellent linearity was observed for the analyses between peak areas and concentrations over the range tested (R^2^ = 0.9999). The limit of detection (LOD) and limit of quantitation (LOQ) of the method were evaluated considering an analyte concentration that would yield an *S*/*N* value of 3 and 10. These values were experimentally verified by injecting standard solutions of the compounds at the LOD and LOQ concentrations. The LOD and LOQ values for the target analytes were 0.10 and 0.29 µg/mL respectively.

### 2.3. Precision (Recovery)

Intra- and inter-day variability of the α-asarone from PF extract was measured to validate the precision. Intra-day variability was determined by analyzing the samples within 24 h. The test samples were injected three times and the relative standard deviation (RSD) value was calculated for the concentration of each analyte in the extract. α-Asarone standard solutions were injected three times at concentrations of 50, 100 and 200 μg/mL onto the HPLC system, and RSD values were calculated for the retention time and peak area. As a result, the intra- and inter-day peaks area of the RSDs were 0.44%, 0.06%, 0.36% and 1.64%, 2.80%, 2.16% at 50, 100 and 200 μg/mL concentrations, respectively. The intra- and inter-day retention times of the RSDs for the α-asarone were the ranging of 0.02% and 0.05%, respectively. The percentage recovery values obtained by comparing the results from samples were in a small range, 97.6%–99.2% with RSDs of less than 3.0% (Table 2).

Analysis of polyphenols in natural products is normally carried out by reversed-phase HPLC using a wide variety of mobile phase compositions. In previous studies, reversed-phase HPLC based on C18 stationary phase has been preferred to achieve polyphenol rather than using normal phase [19,20]. In both cases UV detectors have been employed.

### 2.4. Optimization of Extraction Conditions

In general, the extraction efficiency of a compound is influenced by multiple parameters, such as ratio of liquid to raw material (mL/g, X_1_), extraction time (hour, X_2_) and ethanol concentration (X_3_, %), whose effects will be either independent or interactive. Here, the investigation into the extraction of α-asarone from PF was optimized through the coefficients of the regression equation, calculated using statustucak abakysus system (SAS) software (SAS Institude Inc., Cary, NC, USA, version 9.1). In this study, the 17 designed experiments for optimizing the three individual parameters in the current CCD were shown in Table 3.

The regression equations for response surface was listed in Table 4. The coefficient of determination, R^2^, of the model was calculated as 0.9354, suggesting the model explains 93.54% of variability in the data. This result showed that the model adequately represents the relationship between the response and independent variables.

**Figure 2:**
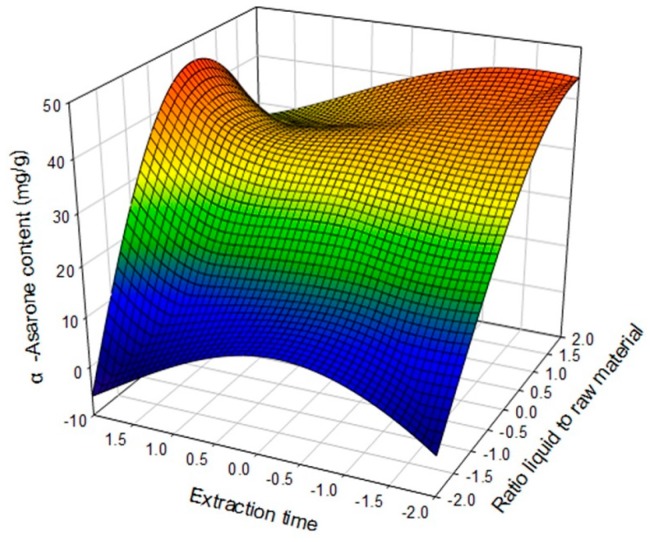
Response surface plot for the effects of ratio of liquid to raw material (X_1_) and extraction time (X_2_) on α-asarone content (mg/g dried plant).

The effect and optimum conditions of processing parameters on the content and purification factor of α-asarone were obtained by studying the three-dimensional (3D) response surface curves. The plots show the effect of X_1_, X_2_ and X_3_, on the α-asarone content as determined using the extraction method. α-Asarone content increased with X_1_ and X_2_. The highest α-asarone content was observed for extractions longer two hours and samples over the 5 g level (Figure 2).

**Figure 3:**
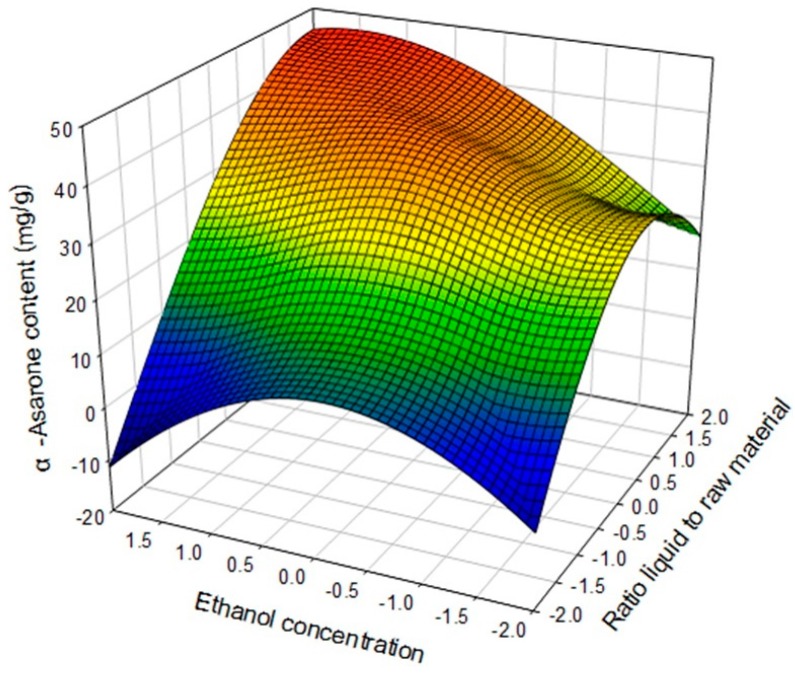
Response surface plot for the effects of ratio of liquid to raw material (X_1_) and ethanol concentration (X_3_) on α-asarone content (mg/g dried plant).

Figure 3 showed the effect of X_1_, X_3_, and their mutual interaction on α-asarone extraction at a reaction temperature of 70 °C. At a high ratio of liquid to raw material (+2), with high ethanol concentration (+2), the content was 50.0 mg/g level.

**Figure 4:**
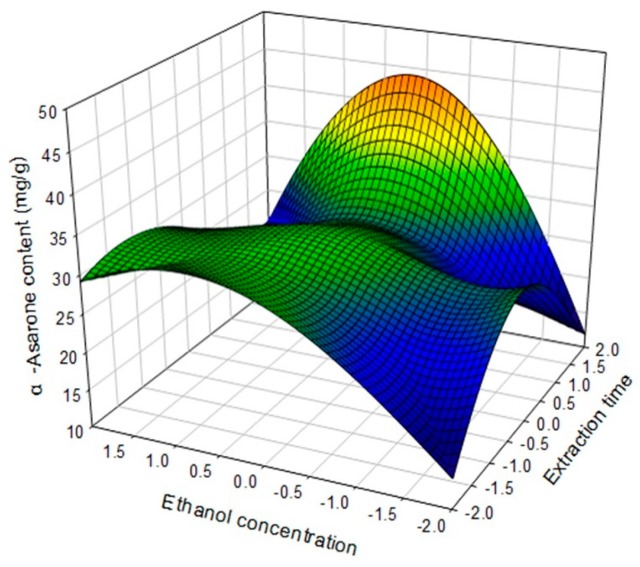
Response surface plot for the effects of ratio of extraction time (X_2_) and ethanol concentration (X_3_) on α-asarone content (mg/g dried plant).

However, a low ratio liquid to raw material (−2) and low ethanol concentration (−2) led a lower content of 9.0 mg/g. Investigation of the mutual effects of X_2_ and X_3_ on the content of α-asarone showed it decreasing with decreasing X_2_ and X_3_ levels (Figure 4).

Overall, a larger content of α-asarone was observed by both increasing the ratio of liquid to raw material and extraction time. This information was used to test the accuracy of the model’s prediction of optimum response values by comparing it with the optimum levels obtained by the RSM optimization. Table 5 showed that the predicted values are close to the experimental values. The predicted peak point led to an optimization content of 40.56 mg/g α-asarone with the corresponding independent parameters being: extraction time of 1.76 h, extraction sample weight of 13.5 g, and extraction solvent concentration of 90.37%. The optimization study was performed in order to verify the suitability of the equation model used for this prediction. Under the optimal conditions, the experimental content of α-asarone was 43.84 mg/g; close to the predicted value. Based on these data, the extraction process was optimized to obtain desirable responses at maximum.

RSM is an optimization tool that is being adopted by development studies of food and natural extract. RSM has been successfully applied to various areas of food engineering and biotechnology, including optimizing extractions of bioactive compounds [21], citric acid production [22], polysaccharide extraction [23] and polyphenols extraction in tea [24]. The efficiency of an extraction process was influenced by factors such as solvent composition, extraction temperature, extraction time, and solvent to solid ratio [25]. These applications include: Ambati and Ayyanna’s optimum fermentation conditions for extraction of citric acid, using substrate concentration, potassium ferrocyanide, and ammonium nitrate, from Palmyra jiggery [22]; and the extraction of oil from *Juniperus communis* needles investigated using supercritical CO_2_ pressure, extract temperature and time [26]. Recently, Wani et al. reported optimum conditions (R^2^ = 0.8057) for extraction of total polyphenols from dried apricot fruit extracts [27]. In this study, a varying level of solvent-to-solid ratio did influence α-asarone content. In addition, in some published studies suggested that this parameter could drastically affect the content during the extraction of components from food and natural sources.

## 3. Materials and Methods

### 3.1. Plant Material, Extraction and Isolation

The dried PF purchased from a local market in Chuncheon, Korea. The voucher sample (RIC-2012-5) has been deposited at the Center for Efficacy Assessment and Development of Functional Foods and Drugs, Hallym University, Chuncheon, Korea. The dried PF (100.0 g) were ground and extracted with 80% ethanol at room temperature. The 80% ethanol extract (3.0 g) was subjected to silicagel column chromatography for further with hexante and ethyl acetate gradient system (4:4 to 1:1) to yield α-asarone.

### 3.2. General Experimental Procedures

HPLC was performed using an Agilent Technologies modular model 1100 system (Agilent, Sunnyvale, CA, USA), consisting of a vacuum degasser (G1322A), a quaternary pump (G1311A), an auto-sampler (G1313A), a thermostatted column compartment (G1316A) and a diode array detection (DAD, G1315B) system. ^1^H- and ^13^C-NMR spectra and correlation NMR spectra such as COSY, HMBC, HMQC, and DEPT were obtained from an Avance DPX 400 spectrometer (Bruker, Berlin, Germany) for identification of the isolated compound. These were obtained at operating frequencies of 400 MHz (^1^H) and 100 MHz (^13^C) with (CD_3_)_2_SO and TMS was used as an internal standard. All solvents used in the analysis were HPLC grade obtained from J.T. Baker (Phillipsburg, NJ, USA).

### 3.3. HPLC Analysis

The sample was analyzed on an Eclipse extra dense bonding (XDB)-phenyl column (150 mm × 4.6 mm, 3.5 μm, Agilent). The mobile phase, consisting of 0.1% trifluoroacetic acid (A) and methanol (B) were used at a flow rate of 0.7 mL/min. The gradient elution program was: programmed to reach 50% B at 15 min using a linear gradient, followed by 100% B-line from 15–23 min. The injection volume was 10 μL, and the detection wavelength was 254 nm. The column temperature was maintained at 30 °C using a temperature controller.

### 3.4. Method Validation of α-Asarone from PF

Stock solution of PF Extract was prepared by dissolving in HPLC grade methanol to final concentration of 5000 μg/mL in a 2.5 mL tube. A stock solution of α-asarone was prepared in methanol by weighing 5000 μg of α-asarone standard in a 10 mL volumetric flask. The standard solutions were stored at 4 °C in clear glass volumetric flasks. α-Asarone solution concentrations used for the calibration curves were 10–100 μg/mL. Calibration curves were prepared and assayed in triplicate on three different days to evaluate the LOQ, LOD, selectivity, and stability.

### 3.5. Extraction Process 

The dried PF (1.0 g) was accurately weighed and placed in a capped tube and mixed with 10 mL of 80% ethanol. After wetting the plant material, the tube containing the suspension was immersed at 70 °C in a water bath and irradiated for the predetermined for 30 min. After extraction, the sample was centrifuged at 5000 rpm for 3 min. The supernatant was collected and evaporated under reduced pressure at 40 °C. Samples (5 mg/mL) were dissolved in methanol and then filtered through a 0.45-μm syringe filter before analysis. The α-asarone content of the PF extracts was expressed as mg/g dried plant. The reported data are the combined results of three replications.

### 3.6. Experimental Design for RSM

Optimized extraction conditions for α-asarone were obtained from RSM. A CCD consisting of 17 experimental runs was employed including four replicates at the center point. All runs were carried out in duplicate. CCD was a response surface design requiring five points, coded as −2, −1, 0, +1, and +2. The design variables were X_1_, X_2_, and X_3_. The dependent variable was the α-asarone content. The model equation for the response (Y) to the three independent variables (X_1_, X_2_ and X_3_) is given in the following equation:

$$Y=\beta_{0}+\overset{2}{\underset{i=1}{\sum }}\beta_{i}X_{i}^{\;}+\overset{2}{\underset{i=1}{\sum }}\beta_{ii}X_{i}^{2}+\overset{\;}{\underset{i=1}{\sum }}\;\overset{\;}{\underset{j=i+1}{\sum }}\beta_{ij}X_{i}X_{j}$$

### 3.7. Data Analysis

All calculations and analyses were performed using statistical analysis system (SAS, version 8.0) software and comparisons among data were carried out using one-way analyses of variance, as appropriate. Mean values were considered significantly different when *p* < 0.05. Sigma plot (Systat Software Inc., San Jose, NC, USA, version 11) was used to perform data graphing.

## 4. Conclusions

A simple, rapid and accurate HPLC method was developed and validated for quantification of α-asarone obtained from PF. The application of this method allowed verification of the factors affecting the presence of α-asarone, and determination of the best method for sample processing. The method showed high sensitivity and selectivity, and allowed the α-asarone content of PF extract to be determined. Additionally, RSM was successfully employed to optimize extraction of α-asarone from PF. Optimal conditions for maximum extraction of α-asarone were determined for the controlling factors: ratio of liquid to raw material (13.5 g/10 mL), extraction time (1.76 h) and ethanol concentration (90.37%).

## Figures and Tables

**Table 1:** Statistical analysis for the calibration curves of α-asarone (*n* = 3).

Compound	Slope	Intercept	R^2 1^	LOD ^2^ (μg/mL)	LOQ ^3^ (μg/mL)
α-Asarone	5995.91	−116.94	0.9999	0.10	0.29

^1^ R^2^, correlation coefficient for the 5 data points in the calibration curves (*n* = 3); ^2^ LOD, limit of detection (µg/mL^−1^, *S*/*N* = 3); ^3^ LOQ, limit of quantification (µg/mL^−1^, *S*/*N* = 10).

**Table 2:** Intra and inter day precision data for the retention time and peak area of α-asarone.

Concentration (μg/mL)	Precision				Recovery (%, Mean ± RSD, *n* = 3)
	Intra-Day (*n* = 3)		Inter-Day (*n* = 3)		
	*R*_t_^1^	Area ^2^	*R*_t_	Area	
200	0.02	0.36	0.05	2.16	97.7 ± 1.28
100	0.02	0.06	0.01	2.80	97.6 ± 2.26
50	0.02	0.44	0.02	1.64	99.2 ± 1.23

^1^ Relative standard deviation of retention time (% RSD); ^2^ Relative standard deviation of peak area (% RSD).

**Table 3:** Experimental range and values of the independent variables in the central composite design for optimization of extraction conditions.

No.	X_1_^1^	X_2_^2^	X_3_^3^	α-Asarone Content (mg/g Dried Plant)	
				Experimental	Predicted
1	−1 (5)	−1 (1.5)	−1 (70)	17.47 ^j^	17.45
2	−1 (5)	−1 (1.5)	1 (90)	14.57 ^k^	13.38
3	−1 (5)	1 (2.5)	−1 (70)	19.36 ^i^	21.61
4	−1 (5)	1 (2.5)	1 (90)	14.74 ^k^	14.97
5	1 (13)	−1 (1.5)	−1 (70)	30.93 ^f,g^	33.49
6	1 (13)	−1 (1.5)	1 (90)	46.36 ^a^	40.51
7	1 (13)	1 (2.5)	−1 (70)	31.77 ^f^	35.33
8	1 (13)	1 (2.5)	1 (90)	36.80 ^d^	39.78
9	−2 (3)	0 (2)	0 (80)	7.51 ^l^	6.81
10	2 (15)	0 (2)	0 (80)	39.71 ^c^	37.45
11	0 (10)	−2 (1)	0 (80)	31.71 ^f^	34.39
12	0 (10)	2 (3)	0 (80)	40.82 ^b^	37.24
13	0 (10)	0 (2)	−2 (50)	27.16 ^h^	24.83
14	0 (10)	0 (2)	2 (100)	30.73 ^g^	32.32
15	0 (10)	0 (2)	0 (80)	31.07 ^f,g^	33.59
16	0 (10)	0 (2)	0 (80)	36.37 ^d^	33.59
17	0 (10)	0 (2)	0 (80)	34.31 ^e^	33.59

^1^ X_1_, Ratio of liquid to raw material (mL:g); ^2^ X_2_, Extraction time (hour); ^3^ X_3_, solid-liquid ratio (%). Different letters in rows show statistically significant differences, *p* < 0.05.

**Table 4:** Polynomial equations calculated using the RSM program for extraction conditions.

Response Variable	Second Order Polynomial Equations	R^2^	*p*-Value
α-Asarone (mg/g dried plant)	Y_α-Asarone_ = 31.067841 + 13.243971X_1_^a^ + 2.368721X_2_^b^ + 2.013598X_3_^c^ − 9.165851X_1_^2^ − 1.740172X_1_X_2_ + 2.234780X_2_^2^ + 10.400973X_1_X_3_ + 3.200789X_2_X_3_ − 4.446175X_3_^2^	0.9354	0.001

^a^ X_1_, Ratio of liquid to raw material (mL:g); ^b^ X_2_, Extraction time (hour); ^c^ X_3_, solid-liquid ratio (%).

**Table 5:** Predicted and experimental values of the response at optimized conditions.

Responses Variable	Optimum Extraction Conditions			Values		Morphology
	X_1_^1^	X_2_^2^	X_3_^3^	Predict	Experimental	
α-Asarone content (mg/g dried plant)	10:13.5	1.76	90.37	40.56 ^b^	43.84 ^a^	Saddle point

^1^ X_1_, Ratio of liquid to raw material (mL:g); ^2^ X_2_, Extraction time (hour); ^3^ X_3_, solid-liquid ratio (%). Different letters in rows show statistically significant differences, *p* < 0.05.

## Supplementary Materials

Supplementary materials are available online.
