# Supplementary material for: Rapid High Performance Liquid Chromatography Determination and Optimization of Extraction Parameters of the α-Asarone Isolated from Perilla frutescens L
Source: Molecules. 2017 Feb 10;22(2):270. doi: 10.3390/molecules22020270 (PMC6155921; doi:10.3390/molecules22020270)
Supplement: Supplementary file 1 [file molecules-22-00270-s001.pdf]

# Supplementary Materials: Rapid High Performance Liquid Chromatography Determination and Optimization of Extraction Parameters of the $\alpha$-Asarone Isolated from *Perilla frutescens* L.

**Seung Hwan Hwang, Shin Hwa Kwon, Young-Hee Kang, Jae-Yong Lee and Soon Sung Lim**

**Table S1.** Experimental range and values of the independent variables in the central composite design for optimization of extraction conditions.

| Variables | Symbol Coded | Range and Levels | | | | |
|---|---|---|---|---|---|---|
| | | **−2** | **−1** | **0** | **+1** | **+2** |
| Ratio of liquid to raw material (mL:g) | $X_1$ | 10:3 | 10:5 | 10:10 | 10:13 | 10:15 |
| Extraction Time (h) | $X_2$ | 1 | 1.5 | 2 | 2.5 | 3 |
| Ethanol concentration (%) | $X_3$ | 50 | 70 | 80 | 90 | 100 |
